# Volcanic forcing of global climate cooling at the Younger Dryas onset preserved in North American sediments

**DOI:** 10.1126/sciadv.aec9030

**Published:** 2026-04-29

**Authors:** Lucien Nana Yobo, Alan D. Brandon, Sydney O’Brien, Jessi J. Halligan, Michael R. Waters

**Affiliations:** ^1^ Department of Geology and Geophysics, Texas A&M University, College Station, TX, USA.; ^2^ Department of Geological Sciences, New Mexico State University, Las Cruces, NM, USA.; ^3^ Center for the Study of the First Americans, Department of Anthropology, Texas A&M University, College Station, TX, USA.

## Abstract

The Younger Dryas (YD; ~12.9 to 11.7 thousand years) marks an abrupt return to near-glacial conditions during the last deglaciation, yet its cause remains debated. One possible scenario, the YD impact hypothesis, proposes an extraterrestrial trigger. However, growing geochemical and stratigraphic evidence points toward a volcanic origin. This study presents the ^187^Os/^188^Os isotope and highly siderophile element (HSE) data from the Page-Ladson (PL) site (8JE591) in Florida, a well-dated, continuous sedimentary record spanning the YD onset. The onset in the PL profile is marked by unradiogenic osmium coincident with elevated Os and Re concentrations and a Cl-chondrite–normalized HSE pattern with a compositional range and signature closely matching volcanic aerosol patterns. When integrated with comparable records from Hall’s Cave and the Debra L. Friedkin site in Texas, the unradiogenic ^187^Os/^188^Os ratios align across multiple depositional environments and correlate with a cluster of major bipolar volcanic eruptions (~12.98 to 12.87 thousand years) documented in Greenland and Antarctic ice cores whose cumulative radiative forcing exceeds the most volcanically active intervals of the Common Era. The magnitude and hemispheric asymmetry of this volcanic activity imply forcing of sufficient magnitude capable of disrupting the Atlantic Meridional Overturning Circulation and triggering rapid Northern Hemisphere cooling. These findings provide multiproxy, regionally consistent evidence for a volcanically driven perturbation at the onset of the YD, offering a robust alternative to impact-based explanations.

## Teaser

Osmium isotope from multiple North American sites link Younger Dryas onset to short-lived cluster of major volcanic eruptions.

## INTRODUCTION

**Fig. 1.**
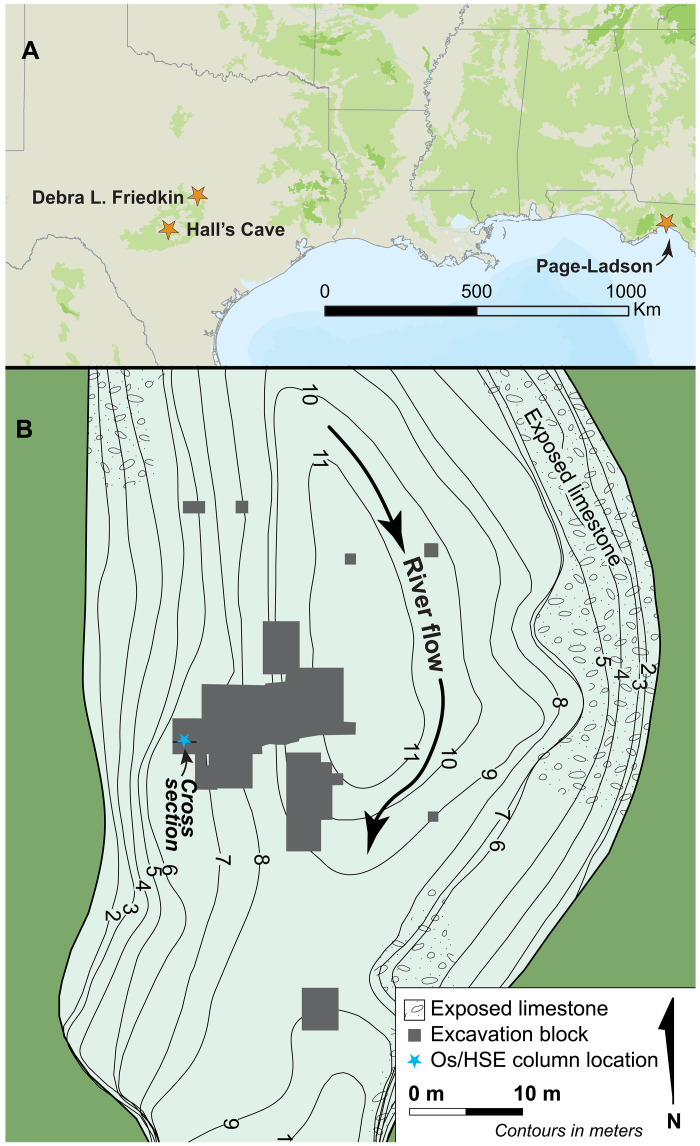
PL site map. (**A**) Location of sample column, also shown are the locations of Hall’s Cave and Debra L. Friedkin. (**B**) Map of the underwater excavations at the PL site.

The cause of the abrupt climate reversal known as the Younger Dryas (YD), from approximately 12.9 to 11.7 thousand years (ka), remains one of the most intense debates of the Late Quaternary (*1*). Characterized by a rapid return to near-glacial conditions during the last deglaciation, the YD is traditionally interpreted in terms of changes in North Atlantic circulation, particularly disruptions to the Atlantic Meridional Overturning Circulation (AMOC) following meltwater input (*2*–*6*). However, over the past two decades, proponents of the YD impact hypothesis (YDIH) have proposed an extraterrestrial impact event as the trigger for this climate anomaly, supposedly coincident megafaunal extinctions, and cultural transitions in North America (*7*–*17*). These claims are challenged by an extensive body of multidisciplinary evidence highlighting fundamental flaws in the stratigraphy, dating, geochemistry, and reproducibility of the purported impact proxies [(*1*) and references therein]. In a recent comprehensive reassessment of the YDIH, Holliday *et al.* (*1*) systematically deconstructed the empirical foundation of the impact hypothesis as well as demonstrated from existing published data that there is no consistent suite of diagnostic impact markers such as shocked minerals, impact structures, or uniquely extraterrestrial geochemical signatures that are convincingly identified and reproduced at the onset of the YD (*1*). Holliday *et al.* (*1*) also asserted that many of the so-called proxies (e.g., microspherules, nanodiamonds, and platinum anomaly) are either misidentified, nonunique, or are better explained by other terrestrial processes (*1*, *16*). Furthermore, the proposed extraterrestrial origin of the Pt anomaly from Greenland (*10*), often cited as support for the YDIH, is not without contention (*18*). High-precision speleothem records synchronized with the Greenland ice-core record places the Pt anomaly 50 years earlier than the onset of the YD (*5*). This offset undermines a causal link between the Pt anomaly and the initiation of the YD, instead suggesting that the anomaly represents an unrelated event, further weakening the impact hypothesis as an explanation for YD onset (*18*). In addition, highly siderophile element (HSE: Os, Ir, Ru, Pt, Pd, and Re) concentrations, typically abundant in extraterrestrial materials, and ^187^Os/^188^Os ratios, show no evidence of an extraterrestrial input in any of the investigated locations thus far (*19*–*22*). Instead, two well-dated sites, Hall’s Cave (*21*) and the Debra L. Friedkin site (*22*) in Texas, USA, have HSE and osmium isotope (^187^Os/^188^Os) ratios showing patterns consistent with a volcanic origin at the onset of the YD rather than extraterrestrial input, further challenging the YDIH. The presence of volcanogenic HSEs in distal continental settings supports the idea that volcanic emissions may have had a broader spatial impact than previously recognized, leaving behind chemically resolvable fingerprints in the sedimentary record. Baldini *et al.* (*23*) identified a large sulfate spike in the Greenland Ice Sheet Project 2 (GISP2) ice core that aligns precisely with the onset of the YD, pointing to a volcanic trigger. This interpretation is further supported by radiogenic isotopes and HSE signatures from Hall’s Cave and the Debra L. Friedkin site (*21*, *22*). In addition, synchronized sulphur isotope records from Greenland and Antarctic ice cores reconstruct an ~110-year interval of heightened volcanic activity between ~12.98 and 12.87 ka before the present (B.P.), featuring four major bipolar eruptions (*24*, *25*). The cumulative stratospheric sulphate burden and radiative forcing during this short interval exceeded that of any known period in the Common Era, indicating a substantial potential for large-scale climatic disruption, particularly in the Northern Hemisphere, where asymmetrical aerosol loading could have suppressed temperatures and perturbed the AMOC (*24*, *25*). Although sustained volcanism is lacking throughout the entirety of the YD interval, such a front-loaded volcanic cluster may have acted as powerful initial forcing under already sensitive boundary conditions. Together, these lines of evidence point to an underappreciated role for volcanism, not necessarily as the sole driver of the YD but as a plausible and testable trigger mechanism. When considered alongside oceanic and cryospheric feedback, a volcanically forced perturbation offers a coherent alternative to impact scenarios and merits further investigation across a more geographically distributed area. Therefore, in this study, we present ^187^Os/^188^Os data from the Page-Ladson (PL) site in Florida (Fig. 1; 8JE591) (*26*–*29*), a well-stratified, radiocarbon-dated paleoenvironmental sequence in northern Florida that spans the onset and duration of the YD interval as a test to both the volcanic- and impact-driven hypotheses. Unlike many previously investigated locales where sampling has been discontinuous or temporally restricted to the presumed “impact layer” [c.f., (*30*)], the PL sequence offers continuous deposition and an established paleoecological framework, including evidence of Late Pleistocene megafauna and early human presence (*26*, *28*). These results are combined with two other North American sites, Hall’s Cave and Debra L. Friedkin (*21*, *22*), to provide a comprehensive assessment of the driver during the onset of the YD in North America. The ^187^Os/^188^Os isotope systematics provide a robust means of fingerprinting source inputs, particularly via shifts in ^187^Os/^188^Os ratios that differentiate unradiogenic mantle-derived signatures from crustal or extraterrestrial sources (*31*, *32*). Our objective here is to test whether large-scale crustal activity in North America, versus an extraterrestrial impact, contributed to geochemical anomalies and regional environmental perturbations during the YD onset. In doing so, we offer a robust explanation grounded in a well-characterized isotopic tracer and contribute to the expanding interpretive framework for YD climate dynamics.

### Geological background and stratigraphy of PL site

**Fig. 2.**
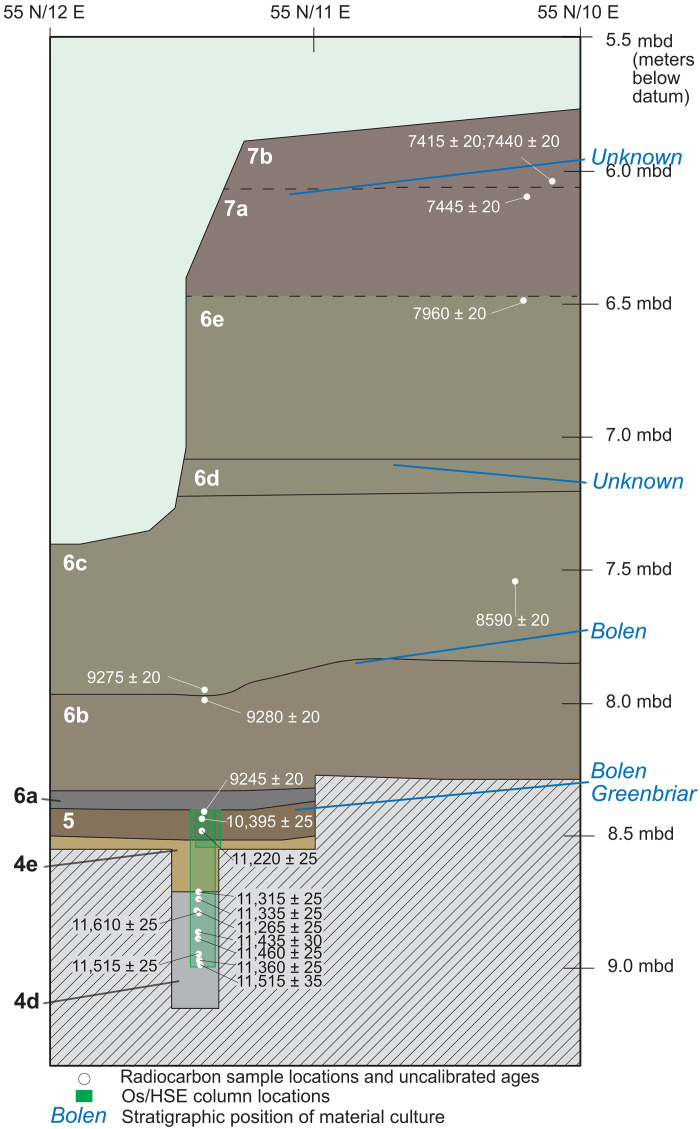
Stratigraphic profile of the south wall of excavation. Excavation units 55 North/10 East and 55 North/11 East, showing location of sample column, radiocarbon ages used in creating age model, and stratigraphic position of cultural material.

The PL site, located in the Half Mile Rise section of the Aucilla River in Florida, is an ~12-m-deep solution sinkhole (doline) that is approximately 30 m north to south and 20 m east to west (Fig. 1). The eastern bank of the site is largely exposed limestone, but the western bank contains sediment deposits that exceed 5 m in thickness and span the latest Pleistocene and Holocene. The Aucilla River flows from southern Georgia into Apalachee Bay with a channel length of only approximately 125 km. The upper Aucilla runs over the Northern Highlands, a highly dissected Miocene delta plain, and is separated from the lower Aucilla by the Cody Escarpment, a probable Sangamon-aged marine terrace (roughly equivalent to the Last Interglacial) (*33*). The lower Aucilla, which contains the PL site, is shallowly incised into the Oligocene-aged Suwannee limestone that makes up the Wakulla Karst Plain (*34*). Site stratigraphy has been previously described (*26*, *35*–*38*). There are eight major stratigraphic units subdivided by changes in color, texture, or contents (Fig. 2 and Table 1) with age ranges indicating episodic deposition from the Last Glacial Maximum (23 ka) until approximately 1 ka. Strata 4d, 4e, and 5 were sampled for this project. All were deposited by water (fluvial or pond processes) and are 5 to 6 m below the current water level in the sinkhole. All contain structure developed by soil formation processes during subaerial exposure. Their model ages are in the chronology section below. Stratum 4d is a dark brown loamy marl with weak subangular blocky structure that contains common whole gastropod shell, common mussel and gastropod shell fragments, common wood fragments, and few subangular blocky limestone pebbles. Stratum 4e, which contains the YD onset at the top, is a gray brown marl that is laminated with alternating silt-sized marl and dissolved organic matter. It has weak subangular blocky structure, few to no gastropods, and very rare preserved wood fragments. It also oxidizes upon exposure. Stratum 5 is a well developed A horizon that developed on an organic stratum (peat) due to wetting and drying of the pond margin. It is a black to dark brown loam with granular to subangular blocky structure and iron and manganese mottling throughout. Stratum 5 contains few to common charred wood fragments and common gastropod lenses. Stratum 5 also contains archaeological material within and on top of it.

**Table 1.** Stratigraphy of the PL site, as refined through 2018 to 2023 excavations. Units 5, 4e, and 4d were sampled for this study. Age ranges for strata were obtained using OxCal version 4.4.4 (*39*) based on published PL ages and ages reported in this paper.

Stratigraphic unit (SU)	Min and max age ka (2σ)	Observed thickness (cm)	Description
8	6.8–1.0	100–130	Highly organic, dark gray brown to black peat with well-preserved leaves and twigs. Occasional laminae of pure sand are present within this stratum. Most of the organics are terrestrial in origin. Abrupt contact with SU 7.
7b	8.3–5.9	40–45	Red woody peat with well-preserved wood fragments. Oxidizes on exposure. Abrupt contact with SU 7a.
7a		40–45	Red moussey peat with few visible organics and common cypress seeds. Oxidizes on exposure. Abrupt contact with SU 6e. Cultural component: Nondiagnostic lithic material at top.
6e	10.5–8.5	40–45	Dark reddish brown organic sapric peat with no shell and rare cypress seeds. Oxidizes on exposure. Abrupt contact with SU 6d.
6d	–	15–20	Dark grayish brown/black sandy silt marl. Little visible shell. Cultural component: Nondiagnostic lithic material at top.
6c	–	50–56	Olive brown very fine sandy silt marl with few fragmentary and complete gastropod shells with few very coarse sand and gravel-sized subrounded calcareous rock fragments. Contact with SU 6b is clear. Oxidizes upon exposure.
6b	10.5–10.4	6–25	Dark olive gray sandy silt marl with common fragmentary and complete gastropod shells and few pieces of preserved organic materials. Abrupt contact with SU 6a. Cultural component: Nondiagnostic lithic material at top.
6a	11.1–10.4	5–7	Olive brown fine sandy silt to silty fine sand marl. Whole and fragmentary gastropod shells are present throughout the unit but are very common in the lower 10 cm. Preserved organic fragments are present but rare throughout. Contact with SU 5 is clear and wavy.
5	12.9–10.8	10–15	Black to dark brown silty clay histosol with granular to subangular blocky structure. Iron and manganese mottling throughout. Highly organic but with few preserved wood fragments. Common gastropod lenses. Lower contact is abrupt and undulatory. Cultural component: Bolen/Greenbriar within and on top
4e	13.1–12.7	20–24	Gray brown silty clay marl. Laminated organic matter, with weak subangular blocky structure. Few to no gastropods, rare preserved wood. Oxidizes on exposure. Abrupt contact with SU 4d.
4d	13.7–12.9	40–50	Dark brown fine sandy silty clay marl. Weak subangular blocky structure. Common mussel and gastropod shell fragments, common preserved wood. Few pea sized limestone pebbles. Weakly oxidizes on exposure. Abrupt contact with SU 4c.
4c	14.2–13.4	40–48	Dark gray brown fine sandy clayey silt marl that does not oxidize upon contact with the water column. Subangular blocky structure. Few gastropods, relatively evenly distributed, and few freshwater mussels, some crushed. Very common tree limbs and trunks of various diameters, often meters long, some burned. Little to no digesta. SU 4c and 4b are separated from each other by a thin (1- to 3-cm-thick) wavy organic lens.
4b	14.4–14.1	45–68	Olive brown fine sandy clayey silt marl that oxidizes to a lighter color upon exposure. Weak medium subangular blocky structure. Gastropod shell common, digesta laminae common. Clear contact with 4a. Cultural component: Pre-Clovis lithic material.
4a	15.0–14.1	0–5	Dark brown sandy clayey silt marl with subangular blocky structure. Gastropod shell and organics including digesta interspersed throughout. Abrupt lower contact with SU 3c. Cultural component: Pre-Clovis lithic material.
3c	15.1–14.1	24–42	Interbedded medium to coarse olive gray quartz sand and well-preserved digesta, which is largely composed of 0.25- to 1.5-cm-diameter twigs cut into 2- to 6-cm lengths, missing bark, and is generally yellow brown to dark yellow brown in color. Sand is typically approximately 30 to 60% by total volume, with occasional sand laminae. Common to many angular limestone pebble and cobble clasts ranging in size from 0.2 to 10 cm. Common tree limbs and large wood fragments (>10 cm in length). Common shell. Clear contact with SU 3b. Cultural component: Pre-Clovis lithic material.
3b	14.9–14.3	0–30	Dark gray sandy digesta. Discontinuous and more compact than overlying 3c and consists of slightly intermixed medium to coarse quartz sands and digesta fragments 2 to 4 cm in length and 0.25 to 1 cm in diameter. Sand percentages are commonly 30 to 60% of volume. Clear wavy contact with Unit 3a. Organic matter, shell, and limestone gravels throughout. Abrupt contact with SU 3a
3a	15.9–14.5	5–25	Light gray medium to coarse quartz sand. Poorly sorted angular limestone gravels and cobbles. Shell common. Little to no digesta but 10 cm + wood fragments and cypress seeds common. Clear to gradual contact with SU 2.
2	18.6–15.5	20+	Red peat, compacted. Common limestone gravel and well-preserved organic matter, largely cypress wood and seeds.
1	23–18.2	10+	Dark brown compact woody peat.

## RESULTS

### Chronology and age model

**Fig. 3.**
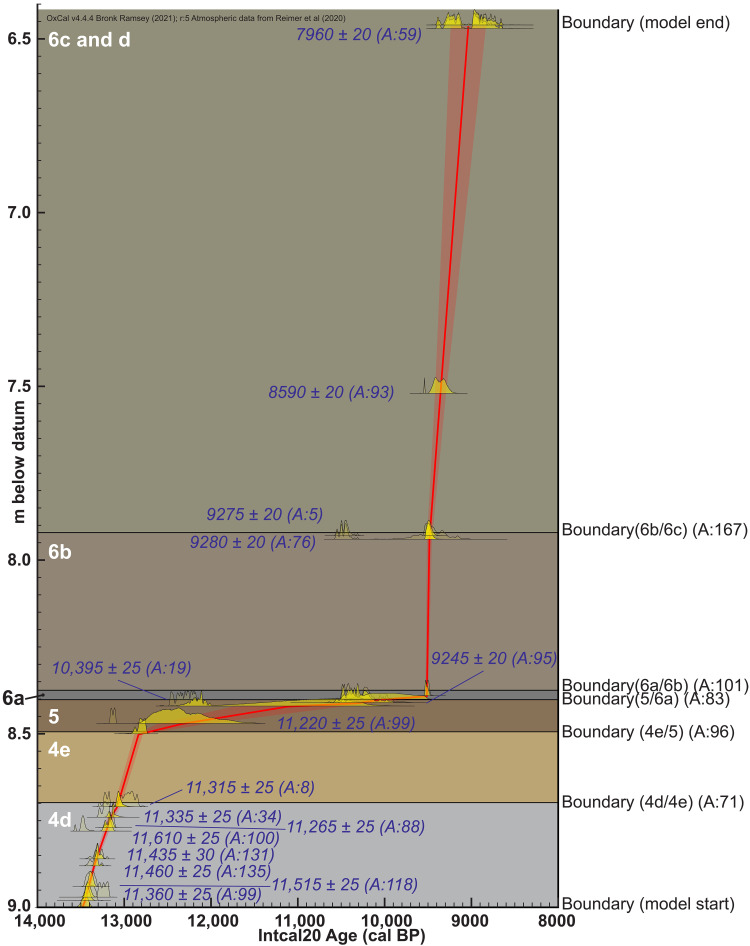
Age model for stratigraphic units 4d to 6d in sample location. Age model is based on dates shown in Fig. 2, with phase model for the entire site (see Strat, Table 1) used to constrain ages of substrata. Radiocarbon ages are plotted by depth, showing model agreement. Unshaded portions of each sample calibration are those the model rejects (*39*, *40*).

PL is among the most thoroughly dated archaeological sites in the Americas, with more than 250 accelerator mass spectrometry radiocarbon ages, most of which were obtained upon short-lived plant matter. The age depth model for this study (Fig. 3) was created via the newly reported ages presented in Fig. 3 and Table 1, using the P-sequence setting within Oxcal’s online version 4.4.4 (*39*), with atmospheric data from Reimer *et al.* (*40*). The model thus includes ages from within 50 cm of the column sample as well as those that were obtained from within the column sample. Several strata had few ages, so the modeled phase ages from the entire dataset were used to confine the start and end of each stratum. We assumed for this phase model, on the basis of the abrupt to clear stratigraphic boundaries between each stratigraphic layer, that one stratum ended deposition before the next stratum was deposited, i.e., that boundaries were sequential. The age depth model was run with two outlier models, one for charcoal, which assumes that all charcoal ages are at least slightly too old, and the general outlier model, which determines the likelihood of fit for each portion of each age’s calibration curve relative to the other ages with the model and the defined phase ages for the stratum as a whole at the site, with a default value probability of any age to be outlier of 0.05 (*41*, *42*). The model output provides an outlier probability table for each age and a model agreement value for each, but Markov chain Monte Carlo simulations will be used to find a best fit for all the model inputs, which means that each model run is likely to provide slightly different values. Stratum 4d spans from a maximum of 13.7 and a minimum of 12.9 ka (at 2σ) based on 13 ages. Stratum 4e, which contains the YD onset, spans from 13.1 to 12.7 ka based on 7 ages. Stratum 5 spans 12.9 to 10.8 ka based on 27 ages. Boundary ages for all strata are presented in Table 2. Model code is presented in the Supplementary Materials.

**Table 2.** Radiocarbon ages from the PL site presented in Fig. 3. Stratum 6e, 6c, 6b, 6a, 5, 4d/4e interface, 4e, 4d, ages were used in age model created in OxCal version 4.4.4 (*39*).

Stratum	Depth below datum	Material	14C age	Laboratory code (UCIAMS)	ID	Fraction modern
7b	6.07	Twig	7415 ± 20	239166	*Taxodium distichum* (Bald cypress)	0.3974 ± 0.0010
7a	6.09	Twig	7445 ± 20	232181	Unidentified hardwood	0.3958 ± 0.0008
7b	6.2	Wood	7740 ± 20	163505	*T. distichum* (Bald cypress)	0.3814 ± 0.0008
6e	6.47	Twig	7960 ± 20	232180	*T. distichum* (Bald cypress)	0.3713 ± 0.0008
6c	7.52	Charcoal	8590 ± 20	232179	*Quercus* (oak)	0.3433 ± 0.0008
6c	7.93	Twig	9275 ± 20	232178	*Quercus* (oak)	0.3153 ± 0.0008
6b	7.94	Wood	9280 ± 20	232177	*Quercus* (oak)	0.3150 ± 0.0007
6a	8.4	Twig	9245 ± 20	232149	Unidentifiable	0.3163 ± 0.0007
5	8.42	Twig	10395 ± 25	232148	*T. distichum* (Bald cypress)	0.2742 ± 0.0007
5	8.47	Wood	11220 ± 25	232147	Unidentifiable - collapsed structure	0.2474 ± 0.0007
4d/4e interface	8.71	Wood	11315 ± 25	232150	*Quercus* (oak)	0.2445 ± 0.0007
4d	8.74	Wood	11335 ± 25	232151	Unidentifiable - collapsed structure	0.2439 ± 0.0007
4d	8.77	Twig	11265 ± 25	232152	*T. distichum* (Bald cypress)	0.2459 ± 0.0007
4d	8.78	Wood	11610 ± 25	232153	*Quercus* (oak)	0.2357 ± 0.0007
4d	8.86	Wood	11435 ± 30	239158	Unidentifiable	0.2409 ± 0.0009
4d	8.88	Twig	11460 ± 25	232154	*T. distichum* (Bald cypress)	0.2402 ± 0.0007
4d	8.94	Twig	11515 ± 25	232155	*Quercus* (oak)	0.2385 ± 0.0007
4d	8.97	Wood	11360 ± 25	232156	Unidentifiable	0.2432 ± 0.0007
4d	8.98	Wood	11515 ± 35	239159	Unidentifiable	0.2385 ± 0.0009

### Osmium isotopes and HSE

**Fig. 4.**
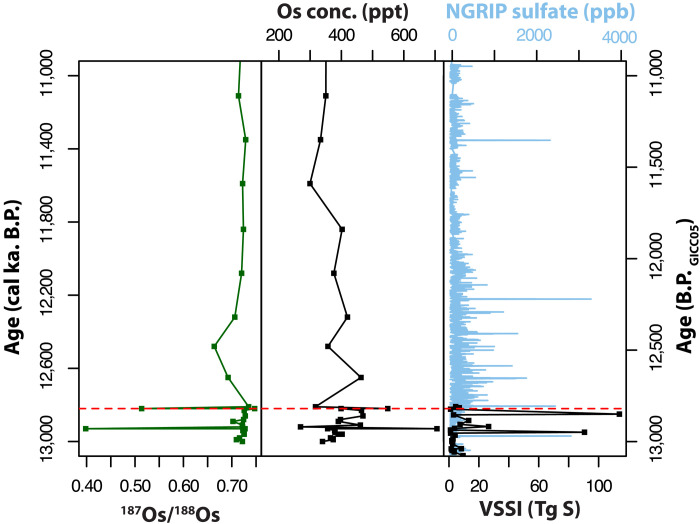
^187^Os/^188^Os and osmium concentration in the stratigraphic profile of the PL site. Red line on the plot indicates the base of the YD. ^187^Os/^188^Os and concentration data (this study). Data of the volcanic stratospheric sulphur injection (VSSI) are from (*24*) and (*80*). The NGRIP sulfate data from (*81*).

The PL ^187^Os/^188^Os ratios range from 0.39 to 0.74. The osmium concentrations range from 165 to 706 parts per thousand (ppt) (Fig. 4). The base of the YD is located at the stratigraphic boundary between the top of unit 4e and the base of unit 5, corresponding to a depth of 8.51 m and dated to 12,820 ± 50 ka B.P. Before the onset of the YD interval, the ^187^Os/^188^Os ratios remain at a consistent background value of 0.72, except for one sample at a depth of 8.6 m, which shows a notable decrease to 0.39 and corresponding osmium concentration of 706 ppt. The base of the YD is marked by a shift to a ^187^Os/^188^Os ratio of 0.51, accompanied by an Os concentration of 550 ppt (Fig. 4). Moving upward through the remainder of unit 4e (from 8.5 to 8.48 m), the ratio gradually decreases from 0.72 to 0.66 and then rises again from 8.47 m upward, returning to the average background ^187^Os/^188^Os value of 0.72. Compared with other sites in North America, the PL site has lower ^187^Os/^188^Os background ratios and consistently higher Os and Re concentrations than the other sites in North America.

**Fig. 5.**
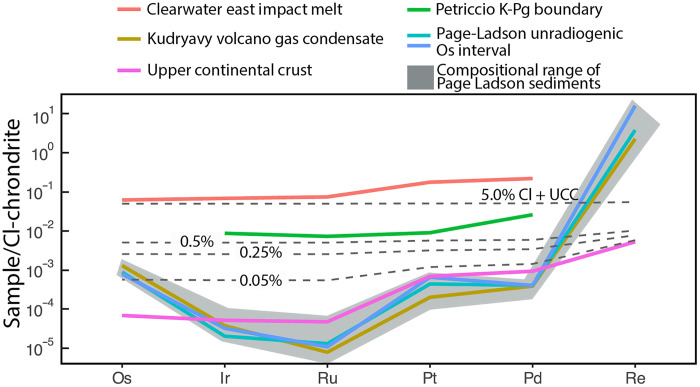
CI chondrite–normalized pattern for unradiogenic osmium isotope interval in PL. The Cl chondrite patterns are shown with an incremental mixing model lines of CI chondrite material into a UCC as well as the envelop range (gray) of CI chondrite–normalized pattern of the PL sediments. UCC values are from Park *et al.* (*82*) and Esser and Turekian (*83*). Average of Kudryavy volcanic gas condensate are from (*43*). Cl chondrite values used for normalization are from Wasson and Kallemeyn (*84*).

The HSE abundances of the PL site are generally similar to those of upper continental crust (UCC; Fig. 5) with exceptions for Ru, Re, and Os. Their Cl chondrite–normalized HSE patterns have a compositional range and signature that is different to UCC because of the higher Os and Re and low Ru concentrations in these PL site sediments. In addition, the HSE patterns for the unradiogenic Os samples show an overlapping compositional pattern to that of volcanic aerosols [Kudryavy Volcano gas condensates (*43*)].

## DISCUSSION

Interpretation of unradiogenic ^187^Os/^188^Os ratios in marine and terrestrial sediments across the YD interval requires caution, given the ongoing debate surrounding the mechanisms responsible for this abrupt climatic event [e.g., (*1*, *17*, *44*)]. While proponents of the YDIH [e.g., (*7*, *8*, *15*, *17*, *45*–*56*)] argue that a chondritic extraterrestrial impact triggered the YD cooling, it is essential to recognize that unradiogenic osmium isotope signatures are not unique to impact events. Similar excursions can also result from terrestrial sources such as increased mantle-derived inputs due to volcanic activity, hydrothermal fluxes, or the weathering of basaltic rocks [e.g. (*57*–*60*)]. These processes can introduce unradiogenic Os into the oceans and atmosphere, yielding geochemical signals that require the addition of other geochemical proxies and stratigraphic context to interpret. With a growing literature (*18*, *19*, *21*, *22*, *61*–*63*) of volcanic source origin for the onset of the YD and the identification of potential episodes of intensified volcanic or tectonic activity in proximity of the YD onset [~12.9 ka; (*23*, *24*)], it is essential to distinguish between extraterrestrial and crustal sources. This distinction requires multiproxy evidence such as HSE enrichments and well-constrained stratigraphic context to clarify the respective contributions of impact versus volcanic processes to the YD event and its associated environmental changes. A Pt or Ir anomaly alone cannot solely be used as evidence of impact as there needs to be a clear enrichment across the suite of HSE’s [e.g., (*64*, *65*)]. A recent study (*18*) found that Pt anomaly as expressed in the Greenland GISP2 ice core was likely a result of local Icelandic fissure volcanism.

**Fig. 6.**
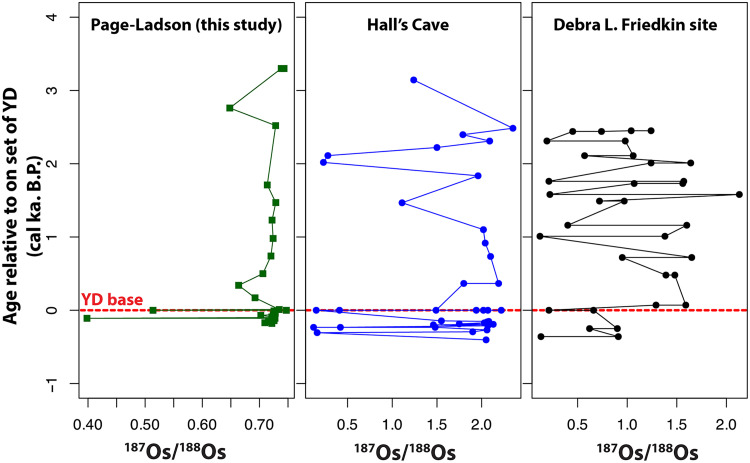
Correlation of the ^187^Os/^188^Os profile from three sites across North America. The YD base is used as a datum. ^187^Os/^188^Os of PL (this study), Hall’s cave from Sun *et al.* (*20*), and Debra L. Friedkin site from Sun *et al.* (*21*). Peaks at the onset of the YD can be correlated across all three site.

The unradiogenic ^187^Os/^188^Os values of 0.5 observed in the PL site are consistent with findings from four other North American locations: Hall’s Cave and Debra L. Friedkin sites in Texas (*21*, *22*) and the Melrose site in Pennsylvania (*20*), which recorded ^187^Os/^188^Os values of 0.15, 0.21, and 0.12 respectively. In the PL site, the two intervals of unradiogenic ^187^Os/^188^Os (≤0.5) at 8.5 and 8.6 cm are not just associated with high osmium concentrations but also higher Re concentrations and have HSE fractionated patterns consistent with enrichment from volcanic aerosols rather than flat normalized patterns that signal increased contributions from ET materials (Fig. 5). However, the background ^187^Os/^188^Os of 0.7 at PL are lower compared to the Hall’s Cave and Debra L. Friedkin values of 2 and 1.5, respectively (Fig. 6). If the higher osmium background ratios of 2 and 1.5 observed at the Hall’s Cave and Debra L. Friedkin sites resulted from a mixture of osmium derived from volcanic aerosol source mixed with a crustal component (*21*, *22*), it is feasible to expect that either the background crustal material at the PL site is composed of lower ^187^Os/^188^Os values of 0.75 or that the enrichment of HSE concentrations may have been influenced by localized processes tied to sulfide mineralization. The sulfides could have been eroded from the nearby sedimentary provenance that supplied material to the PL basin, or, alternatively, they may have precipitated diagenetically within the layers following deposition. However, when the PL record is taken together with the two other records, it is remarkable that the base of the YD in each of these sites is marked by an unradiogenic ^187^Os/^188^Os ratio of 0.15 to 0.5 and below the background values at each of the three locales. This indicates that the Os and HSE anomalies observed in the PL site are not isolated occurrences. Rather, the widespread detection of the unradiogenic ^187^Os/^188^Os at the base of the YD across varied depositional environments ranging from lacustrine to alluvial sediments suggests a regional to potentially continental-scale deposition of HSE-bearing material, likely via aerosols. The spatial consistency of these anomalies implies long-range transport mechanisms. This is the most plausibly stratospheric injection associated with large-scale volcanic eruptions. It also follows that sulfur records from Greenland and Antarctic ice cores show an ~110-year cluster of heightened volcanic activity preceding the YD onset. Abbott *et al.* (*24*) identified that 30 volcanic eruptions including four clusters of major bipolar eruptions, between 12,980 and 12,987 BP_GICC05_, produced a higher volcanic forcing of any comparable interval across the common era. The magnitude and hemispheric asymmetry of these eruptions, three of which predominantly affected the Northern Hemisphere, are particularly relevant as they could have induced rapid and sustained climate perturbations (*24*, *62*, *63*). Such asymmetric radiative forcing would be expected to enhance Northern Hemisphere cooling, amplify cryospheric feedback, and potentially disrupt the AMOC, triggering cascading climate effects consistent with the abrupt onset of the YD. While volcanic eruptions alone may not explain the full 1300-year duration of the YD, the combination of short-term radiative forcing and long-term feedback such as ice sheet regrowth and ocean circulation changes presents a plausible and coherent mechanism for YD initiation. This view aligns with findings expressed in (*23*, *66*, *67*).

**Fig. 7.**
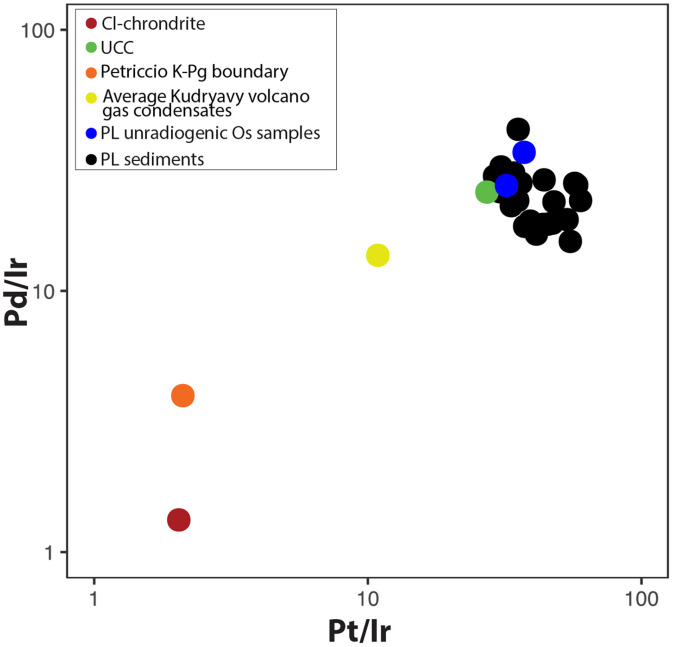
Cross plots of HSE ratio from PL. Pd/Ir versus Pt/Ir plot for the PL sediments (this study) illustrating a nonextraterrestrial source of the signal. UCC values are from Park *et al.* (*82*) and Esser and Turekian (*83*). Average of Kudryavy volcanic gas condensate are from (*43*). Cl chondrite values are from Wasson and Kallemeyn (*84*).

In addition, in the stratigraphic profile of the three sites across North America (Fig. 6), besides the base of the YD, multiple compositionally similar stratigraphic layers exhibiting unradiogenic ^187^Os/^188^Os ratios and elevated HSE concentrations exist. These discrete depositional layers occur at different depths and time layers and do not always match each other. We conclude that they represent separate events, indicating repeated episodes of atmospheric Os/HSE deposition over time. This pattern raises a critical question: What natural process produces multiple, regionally distributed, compositionally consistent Os/HSE enrichments? Large chondritic or iron-rich impacts capable of generating continent-wide geochemical anomalies are exceedingly rare, with estimated recurrence intervals of 100,000 to 1,000,000 years (*68*–*70*). The probability of multiple such impacts occurring over a span of just a few thousand years, each affecting mainly North America and leaving no global signal is implausibly low and, hence, can be ruled out. In contrast, repeated high-magnitude volcanic eruptions, especially those capable of stratospheric injection, are both more frequent and more consistent with the stratigraphic, geochemical, and paleoclimatic data represented in these locales (*21*, *24*, *71*, *72*). There is also a well-documented surge in volcanic activity during deglaciation, with the largest and most abundant eruptive signals occurring between ~17,000 and 6000 years ago as ice sheet retreat reduced lithostatic pressure and enhanced magma production (*73*). The absence of an extraterrestrial contribution to the osmium signal is further reinforced by the Pt/Ir–Pd/Ir cross-plot, in which all PL samples plot well away from fields characteristic of CI chondrites or K-Pg boundary material. Instead, the data form a coherent cluster, indicating a shared geochemical signature that is inconsistent with known extraterrestrial sources (Fig. 7).

Last, the widespread occurrence of Os and HSE anomalies, and their timing alignment with a documented cluster of major volcanic eruptions, provide strong multiproxy support for a volcanically triggered onset of the YD. These findings offer a more robust nonextraterrestrial alternative to the impact hypothesis and underscore the importance of continued integrated geochemical, stratigraphic, and paleoclimatic investigations across North America and beyond. Individual volcanic eruptions would be expected to produce differing patterns of atmospheric and aerosol depositional dispersion such that the varying Os isotope patterns observed between Hall’s Cave, Debra L. Friedkin, and Pennsylvania Melrose through time are consistent with episodic volcanic fallout rather than the uniform, continent-wide distribution anticipated from a large extraterrestrial impact event. The basal YD layers at each site show a similar reduction in ^187^Os/^188^Os, indicating a more concentrated and dispersive volcanic event—one consistent with a large eruption capable of perturbing climate at hemispheric scales. High-resolution ice-core reconstructions (*24*) reveal an ~110-year cluster of heightened volcanic eruptions preceding the YD, with cumulative Northern Hemisphere sulphate forcing exceeding any volcanism of the Common Era. These eruptions, coupled with evidence of volcanogenic HSEs in distal sediments (*13*–*16*), provide a coherent mechanism linking volcanism to abrupt AMOC perturbations and the onset of near-glacial conditions. While volcanism alone does not account for the full duration of the YD (*23*, *74*), its role as a trigger is both plausible and testable, offering a robust alternative to impact-based explanations and underscoring the capacity of clustered eruptions to initiate abrupt climate change. Within this framework, the data in this study present evidence consistent with a volcanic trigger and inconsistent with the YDIH, which, however, does not directly assess a meltwater-driven mechanism.

## MATERIALS AND METHODS

### Samples

In 2019, divers collected two sediment columns from an exposed profile wall at the PL site (Figs. 1 and 2). The columns were excavated under Florida Bureau of Archaeological Research 1A32 permit 1718.050. Archaeological materials have been accessioned under that permit number with the Bureau of Archaeological Research in Tallahassee Florida. The sediment profile was carefully cleaned using ceramic tools with an induction dredge to remove waste sediment. The columns were collected using vertically split vinyl storm gutter sections that were pressed vertically into the sediment using the ceramic knives to aid penetration. Once the gutter was fully seated, the top of the section was exposed, plastic fishing line was used to cut the sediment away from the profile wall, and the other half of the gutter was slid over the exposed back side of the column, overlapping the front gutter. The whole sample was duct-taped and raised to the surface maintaining orientation. In the Underwater Geoarchaeology Laboratory, the gutter sections were separated, and each column was cleaned with glass slides and sampled using ceramic tools in 1-cm increments. Each sample was collected and stored in a 4-mm plastic bag for transport to Texas. Columns overlapped through stratum 5, so there are multiple samples from the same elevation.

### Os isotopes and HSE

Thirty-one samples from column samples 36 and 37, recovered from the PL site (Fig. 2 and table S1), were processed for HSE and ^187^Os/^188^Os isotope analysis at the Thermal Ionization Mass Spectrometry Laboratory, University of Houston. Samples were processed following procedures previously described in Sun *et al.* (*21*, *22*) and Nana Yobo *et al.* (*75*). Briefly, each sample was dried, powdered in an alumina ceramic mortar (i.e., metal-free processing), and homogenized to minimize the nugget effect, a known issue that may influence ^187^Os/^188^Os ratios in sediments [e.g., (*21*)]. One gram of each homogenized sample was weighed and placed in individual Carius tubes (CT), which were then placed in a dry ice–ethanol bath to prevent osmium volatilization during spike and acid addition. A mixed enriched spike containing ^99^Ru, ^105^Pd, ^185^Re, ^190^Os, ^191^Ir, and ^194^Pt was added to each sample. This was followed by the addition of inverse aqua regia to the CTs after which they were sealed. After sealing, the CTs were then placed in the oven at 240°C for 48 hours. Following digestion, the CTs were removed from the oven and the Os extracted from the solution using CHCl_3_ followed by back-extraction into 9N HBr, as described in (*76*). The HBr solutions containing Os were dried down, and the residues were further purified by microdistillation with a CrO_3_H_2_SO_4_ solution and collected in 9N HBr. The Os isotope compositions were determined using a Thermo Fisher Scientific Triton Plus, thermal ionization mass spectrometer in negative mode, with a secondary electron multiplier using peak hopping mode at the University of Houston. The long-term average and precision of ^187^Os/^188^Os for the University of Maryland (UMD) Os standard using the SEM is 0.11380934 ± 0.00033913 (2 SD) with *N* = 49 and within error of the accepted value of 0.1138067 ± 0.000021 (*77*). The residual solutions after Os removal, containing Re and all other HSE, were dried at 80°C. The dried residues were dissolved in 6N HCl and again taken to dryness. This step was repeated twice to ensure full conversion to the chloride form. The sample solutions were then passed through Eichrom AG 1X8 100–200 mesh anion resin following a modified procedure from Day *et al.* (*78*). The collected HSE and Re fractions were dried down and then converted to chloride form by redissolving in HCl. The Re fractions were then further purified using Eichrom AG50X-8 100–200 mesh cation resin following the procedure of Puchtel and Humayun (*79*). After purification, the HSE isotopes were measured on the Element XR at the Williams Radiogenic Laboratory at Texas A&M University. This ensures minimization of isotopic interferences while maximizing signal intensities. Mass fractionation factors were determined by sample/standard bracketing using a standard of natural isotopic composition and then applying the obtained exponential mass fractionation factor to the sample measurements. Average of procedural blanks (*N* = 9) were 6.9, 2.37, 8.59, 6.54, 19, and 0.29 pg for Ir, Ru, Pt, Pd, Re, and Os, respectively.

## Supplementary Materials

### This PDF file includes:

Supplementary Text of the OxCal Age Depth Model Code Tables S1 and S2
