# Supplementary material for: Volcanic forcing of global climate cooling at the Younger Dryas onset preserved in North American sediments
Source: Sci Adv. 2026 Apr 29;12(18):eaec9030. doi: 10.1126/sciadv.aec9030 (PMC13127594; doi:10.1126/sciadv.aec9030)
Supplement: Supplementary file 1 — Supplementary Text of the OxCal Age Depth Model Code Tables S1 and S2 [file sciadv.aec9030_sm.pdf]

Science Advances
AAAS

Supplementary Materials for

Volcanic forcing of global climate cooling at the Younger Dryas onset preserved in North American sediments

Lucien Nana Yobo *et al.*

Corresponding author: Lucien Nana Yobo, lnanayobo@tamu.edu

Sci. Adv. **12**, eaec9030 (2026)
DOI: 10.1126/sciadv.aec9030

This PDF file includes:

Supplementary Text of the OxCal Age Depth Model Code
Tables S1 and S2

Supplementary Text

OxCal Age Depth Model Code

```
Options()
 {
  SD1=TRUE;
  Curve="intcal20.14c";
  SD2=TRUE;
 };
 Plot()
 {
  Outlier_Model("General",T(5),U(0,4),"t");
  Outlier_Model("Charcoal",Exp (1,-1 0,0),U(0,3),"t");
  P_Sequence("rare earth",1)
  {
   boundary ("bottom 4d")
   {
    z=900;
   };
   R_date("239159", 11515, 35)
   {
    z= 898;
    Outlier("General",0.05);
   };
   R_date("232156", 11360, 25)
   {
    z= 897;
    Outlier("General",0.5);
   };
   R_date("232155", 11515, 25)
   {
    z= 894;
    Outlier("General",0.05);
   };
   R_date("232154", 11460, 25)
   {
    z= 888;
    Outlier("General",0.05);
   };
   R_date("239158", 11435, 30)
   {
    z= 886;
    Outlier("General",0.05);
   };
```

```
R_date("232153", 11610, 25)
{
 z= 878;
 Outlier("General",0.5);
};
R_date("232152", 11265, 25)
{
 z= 877;
 Outlier("General",0.5);
};
R_date("232151", 11335, 25)
{
 z= 874;
 Outlier("General",0.05);
};
R_date("232150", 11315, 25)
{
 z= 871;
 Outlier("General",0.05);
};
boundary ("top 4d/bottom 4e")
{
 R_Date("model 4e base",11019,40);
 z=871;
};
boundary ("top 4e/bottom 5")
{
 R_Date("model 4e top",10880,51);
 z=850;
};
R_date("232147", 11220, 25)
{
 z= 847;
 Outlier("General",0.5);
};
R_date("232148", 10395, 25)
{
 z= 842;
 Outlier("General",0.05);
};
Boundary ("top 5 bottom 6")
{
 R_Date("model 5 top",9047,102);
 z=841;
};
R_date("232149", 9245, 20)
```

```
{
 z= 840;
 Outlier("General",0.5);
};
Boundary("end 6a")
{
 R_Date("model end 6a",8502,28);
 z=839;
};
R_date("293403", 9225, 25)
{
 z= 796;
 Outlier("General",0.05);
};
Boundary("Start 6c")
{
 R_Date("model start 6c",8483,126);
 z=794;
};
R_date("232177", 9280, 20)
{
 z= 794;
 Outlier("Charcoal",1);
};
R_date("232178", 9275, 20)
{
 z= 793;
 Outlier("General",0.05);
};
R_date("232179", 8590, 20)
{
 z= 752;
 Outlier("Charcoal",1);
};
R_date("293390", 8255, 25)
{
 z= 650;
 Outlier("General",0.05);
};
R_date("232180", 7960, 20)
{
 z= 647;
 Outlier("General",0.05);
};
Boundary("end of model")
{
```

```
  z=646;
 };
};
};
```

Table S1. Provenience and raw sample data for Os/HSE sample columns.

Column #	Sample #	Actual Depth (mbd)		Column depth (cm)		Stratum	Weight	
		Top	Bottom	Top	Bottom		Wet	Dry
37	1	8.53	8.54	58	59	5	16.86	13.75
37	2	8.51	8.53	56	58	5	16.26	8.36
37	3	8.5	8.51	55	56	5	30.89	19.45
37	4	8.49	8.5	54	55	5	38.17	27.4
37	5	8.48	8.49	53	54	5	28.55	15.21
37	6	8.47	8.48	52	53	5	38.89	25
37	8	8.46	8.47	51	52	5	38.8	25.35
37	9	8.45	8.46	50	51	5	51.01	36.01
37	10	8.44	8.45	49	50	5	39.68	22.08
37	11	8.43	8.44	48	49	5	46.46	29.01
37	12	8.42	8.43	47	48	5	35.35	19.59
37	14	8.41	8.42	46	47	5	34.89	20.71
37	15	8.4	8.41	45	46	5	33.59	22.9
37	17	8.39	8.4	44	45	5	36.93	22.3
36	18	8.39	8.4	1	2	5	29.86	16.21
36	19	8.4	8.41	2	3	5	40.63	23.36
36	20	8.41	8.42	3	4	5	30.97	17.03
36	21	8.42	8.43	4	5	5	33.4	16.7
36	22	8.43	8.44	5	6	5	40.55	23.83
36	23	8.44	8.45	6	7	5	41.49	27.7
36	24	8.45	8.46	7	8	5	44.32	22.27
36	25	8.46	8.47	8	9	5	35.96	17.15
36	26	8.47	8.48	9	10	5	39.19	20.7
36	27	8.48	8.49	10	11	5	38.29	25.86
36	28	8.49	8.5	11	12	5	28.93	13.02
36	29	8.5	8.51	12	13	5/4e	54.86	34.69
36	30	8.51	8.52	13	14	4e	44.87	19.69
36	31	8.52	8.53	14	15	4e	44.22	21.96
36	32	8.53	8.54	15	16	4e	47.36	24.52
36	33	8.54	8.55	16	17	4e	53.63	30.96
36	34	8.55	8.56	17	18	4e	52.86	26.49
36	35	8.56	8.57	18	19	4e	55.62	30.81
36	36	8.57	8.58	19	20	4e	51.35	29.83
36	37	8.58	8.59	20	21	4e	54.6	30.53

Column #	Sample #	Actual Depth (mbd)		Column depth (cm)		Stratum	Weight	
		Top	Bottom	Top	Bottom		Wet	Dry
36	38	8.59	8.6	21	22	4e	55.99	43.55
36	39	8.6	8.61	22	23	4e	47.98	28.38
36	40	8.61	8.62	23	24	4e	43.08	26.35
36	41	8.62	8.63	24	25	4e	42.92	25.9
36	42	8.63	8.64	25	26	4e	34.12	20.5
36	43	8.64	8.65	26	27	4e	37.99	21.5
36	44	8.65	8.66	27	28	4e	37.18	23.22
36	45	8.66	8.67	28	29	4e	45.7	32.14
36	46	8.67	8.68	29	30	4e	38.84	27.94
36	47	8.68	8.69	30	31	4e	39.93	28.8
36	48	8.69	8.7	31	32	4d?	41.14	23.15
36	49	8.7	8.71	32	33	4d?	30.63	17.36
36	52	8.71	8.72	33	34	4d	50.08	28.51
36	53	8.72	8.73	34	35	4d	42.44	24.22
36	55	8.73	8.74	35	36	4d	44.31	25.25
36	56	8.74	8.75	36	37	4d	46.22	26.64
36	58	8.75	8.76	37	38	4d	47.63	27.3
36	59	8.76	8.77	38	39	4d	40.24	23.13
36	61	8.77	8.78	39	40	4d	40.27	25.15
36	63	8.78	8.79	40	41	4d	46.11	31.29
36	64	8.79	8.8	41	42	4d	48.04	36.63
36	65	8.8	8.81	42	43	4d	47.42	42.29
36	66	8.81	8.82	43	44	4d	44.82	25.5
36	67	8.82	8.83	44	45	4d	40.8	22.93
36	68	8.83	8.84	45	46	4d	48.64	27.39
36	69	8.84	8.85	46	47	4d	43.36	24.49
36	70	8.85	8.86	47	48	4d	35.87	26.82
36	72	8.86	8.87	48	49	4d	46.04	36.95
36	73	8.87	8.88	49	50	4d	40.94	26.64
36	75	8.88	8.89	50	51	4d	47.82	31.54
36	76	8.89	8.9	51	52	4d	58.06	35.4
36	78	8.9	8.91	52	53	4d	48.43	28.52
36	79	8.91	8.92	53	54	4d	44.93	25.04
36	81	8.92	8.93	54	55	4d	42.08	24.02
36	83	8.93	8.94	55	56	4d	43.26	27.97
36	84	8.94	8.95	56	57	4d	46.96	27.69
36	85	8.95	8.96	57	58	4d	56.53	31.58
36	87	8.96	8.97	58	59	4d	36.7	21.3

Column #	Sample #	Actual Depth (mbd)		Column depth (cm)		Stratum	Weight	
		Top	Bottom	Top	Bottom		Wet	Dry
36	89	8.97	8.98	59	60	4d	55.12	29.95

Table S2: Osmium isotopes and PGE data from the Page-Ladson section.

Strat-Depth (cm)	Sample number	187Os/188Os	±2s	Os (ppb)	Ir (ppb)	Ru (ppb)	Pt (ppb)	Pd (ppb)	Re (ppb)
8.39	36-17	0.74	0.011	0.4101	0.0093	0.0119	0.3452	0.3314	63
8.39	36-18	0.74	0.014	0.1651	0.0072	bdl	0.2970	0.1159	69
8.4	36-19	0.65	0.007	0.4377	0.0077	bdl	0.3573	0.1383	78
8.41	36-20	0.73	0.007	0.3514	0.0076	0.0008	0.1440	0.1056	76
8.42	36-21	0.71	0.007	0.3504	0.0067	bdl	0.2869	0.1213	64
8.43	36-22	0.73	0.007	0.3334	0.0060	bdl	0.3588	0.1331	87
8.44	36-23	0.72	0.008	0.2996	0.0068	bdl	0.2538	0.1200	74
8.45	36-24	0.72	0.006	0.4025	0.0080	0.0006	0.2840	0.3334	120
8.46	36-25	0.72	0.007	0.3761	0.0078	0.0002	0.4275	0.1205	117
8.47	36-26	0.71	0.006	0.4195	0.0086	0.0013	0.4620	0.1621	133
8.48	36-27	0.66	0.009	0.3571	0.0069	bdl	0.3267	0.1258	121
8.49	36-28	0.69	0.008	0.4628	0.0088	0.0067	0.3869	0.1591	235
8.5	36-29	0.73	0.008	0.3172	0.0061	0.0000	0.2772	0.1099	140
8.51	36-30	0.51	0.003	0.5486	0.0063	bdl	0.3014	0.1381	140
8.51	36-30	0.75	0.011	0.3990	0.0087	0.0083	0.3809	0.2303	143
8.52	36-31	0.73	0.006	0.4645	0.0069	0.0005	0.4009	0.1760	141
8.54	36-33	0.73	0.002	0.4692	0.0107	0.0010	0.3877	0.2770	119
8.56	36-35	0.72	0.003	0.3973	0.0085	0.0061	0.3329	0.1571	134
8.57	36-36	0.70	0.003	0.3903	0.0064	0.0019	0.3623	0.1642	89
8.58	36-37	0.72	0.004	0.4609	0.0081	0.0005	0.2862	0.1800	149
8.59	36-38	0.72	0.003	0.2690	0.0058	0.0037	0.2180	0.1975	120
8.6	36-39	0.40	0.001	0.7060	0.0046	0.0078	0.1379	0.1186	43
8.6	36-39	0.73	0.003	0.3562	0.0137	0.0069	0.5671	0.2268	598
8.61	36-40	0.72	0.003	0.3785	0.0098	0.0177	0.2993	0.2914	211
8.62	36-41	0.72	0.003	0.3797	0.0074	0.0135	0.2258	0.1777	106
8.63	36-42A	0.73	0.003	0.3903	0.0071	0.0125	0.2357	0.1496	99
8.63	36-42B	0.73	0.003	0.4025	0.0078	0.0119	0.2672	0.2217	97
8.64	36-43	0.72	0.003	0.3653	0.0085	0.0157	0.2612	0.2104	92
8.65	36-44	0.71	0.003	0.3739	0.0074	0.0210	0.2380	0.1866	70
8.66	36-45	0.72	0.003	0.3396	0.0072	0.0146	0.2090	0.1983	81
